# Evolving Righteousness in a Corrupt World

**DOI:** 10.1371/journal.pone.0044432

**Published:** 2012-09-12

**Authors:** Edgar A. Duéñez-Guzmán, Suzanne Sadedin

**Affiliations:** 1 Department of Organismic and Evolutionary Biology, Harvard University, Cambridge, Massachusetts, United States of America; 2 Laboratory of Socioecology and Social Evolution, Katholieke Universiteit Leuven, Leuven, Belgium; Hungarian Academy of Sciences, Hungary

## Abstract

Punishment offers a powerful mechanism for the maintenance of cooperation in human and animal societies, but the maintenance of costly punishment itself remains problematic. Game theory has shown that corruption, where punishers can defect without being punished themselves, may sustain cooperation. However, in many human societies and some insect ones, high levels of cooperation coexist with low levels of corruption, and such societies show greater wellbeing than societies with high corruption. Here we show that small payments from cooperators to punishers can destabilize corrupt societies and lead to the spread of punishment without corruption (righteousness). Righteousness can prevail even in the face of persistent power inequalities. The resultant righteous societies are highly stable and have higher wellbeing than corrupt ones. This result may help to explain the persistence of costly punishing behavior, and indicates that corruption is a sub-optimal tool for maintaining cooperation in human societies.

## Introduction

The role of punishment in maintaining cooperative societies has attracted considerable attention from theorists [1]–[6], and their findings may have far-reaching implications for the social sciences. Punishment – inflicting harm on individuals who fail to cooperate [5], [6] – is thought to facilitate cooperation within societies as diverse as those of humans [7]–[9], chimpanzees [10] and insects [11].

However, the evolutionary maintenance of punishment itself presents a problem [5]. Punishment is likely to be costly to punishers: it requires effort, and risks provoking retaliation. Therefore, punishers are likely to be removed by natural selection [5]. In human societies, where cultural evolution is prominent, individuals may also learn to avoid punishing others because of these costs [12], [13].

Models suggest that costly punishment can be maintained if punishers may defect [14]–[16], a scenario termed corruption [17]. Such corruption has been documented among social wasps [17], [18] and ants [19]. Eldakar and Wilson [16] note that defectors have an incentive to punish because doing so increases the proportion of cooperators available to exploit. Allowing punishers to defect can effectively create a division of labor between punishers and cooperative non-punishers, maintaining cooperation in the society as a whole.

In many realistic scenarios, there may be power inequalities between punishers and non-punishers. For example, Úbeda and Duéñez-Guzmán [20] explored the effects of allowing punishers to defect with reduced punishment. They termed this scenario the “corruption game”. The results showed that when power inequalities were small, defecting punishers could help to maintain a cooperative non-punishing population. The model might apply, for example, to the social wasp *Dolichovespula sylvestris*, where punishing behavior appears to be largely confined to defectors and queens [17]. However, in other insect societies, punishment appears to be widespread while defectors are rare, a scenario that we will call righteousness. For example, Kawabata and Tsuji [21] introduced individuals with developed ovaries to pre-existing colonies of the queenless Japanese *Diacamma* sp. ants. They found that such individuals were aggressively attacked by ants with inactive ovaries. Ants are thought to lack the cognitive resources for reputation systems, so the existence of righteousness in these groups presents a puzzle.

Úbeda and Duéñez-Guzmán [20], found that corruption could sometimes increase the net wellbeing of the population (that is, the cumulative payoff of individuals). This occurred because defecting punishers could maintain cooperation in a non-punishing sub-population that would otherwise defect. Úbeda and Duéñez-Guzmán [20] argued that this result provides insight into human psychology, noting that corruption is widespread in many human societies and that individuals increase their moralizing (but not moral behavior) when their power increases [22]. Furthermore, the authors concluded that economic policy may “use corruption to the advantage of a society”, arguing that “the punishment inflicted on [punishers] should always be lower than the punishment inflicted on [non-punishers]” in order to maintain cooperation.

Corruption in human societies carries large and well-documented costs to many aspects of individual and societal wellbeing. Such costs can be measured in terms of social capital [23], [24], happiness and life satisfaction [25], [26], economic development [27]–[31] and health [32], [33]. Given these costs, it is important to establish whether facilitating corruption via power inequalities is indeed a useful tool for maintaining cooperation in human societies.

Empirical evidence provides little support for the idea that corruption assists human cooperation [31]. Inequality does indeed correlate positively with corruption [30]. However, inequality and corruption are also positively related to crime [30] and negatively related to trust [26]. Numerous studies have argued that corruption weakens social networks [23], as well as decreasing investment [27] and sustainable development [31].

Overall, this evidence suggests that corruption undermines both cooperation and wellbeing in human societies. This observation conflicts with the prediction of the corruption game, that cooperation and wellbeing should be greater in societies that permit corruption. In addition, the model does not explain the existence of apparently righteous social insects [21]. It seems the corruption game fails to capture some relevant aspects of punishment.

Úbeda and Duéñez-Guzmán [20] identified globally stable equilibria where the population consisted of a mixture of punishing and non-punishing cooperators. The authors argued that these equilibria were structurally unstable: that is, a small perturbation to the game payoffs could destroy them, and push the population to a different equilibrium. Such perturbations are likely to occur by chance in natural populations. A crucial question, then, is what would be the long-term outcome of such perturbations?

At least one such perturbation appears to be a general feature of human psychology. Costly punishment is used to express negative emotion [34]. Expressions of anger result in increased social status and perceived competency [35], and aggression enhances perceived popularity and social centrality [36]. Anger especially enhances status when it is perceived as retaliatory [37], [38]; in this situation, observers often respond uncritically to hostile action [39], [40] and may even assist punishers [41]. In humans, social status is strongly related to several forms of wellbeing, including health [42], happiness [43], absence of psychological distress [44], and income [45], as well as evolutionary fitness [46]. The increased status of individuals who express anger at injustice can therefore be interpreted as a small payment to punishers.

More generally, the tendency to punish may have social consequences for the punisher beyond the immediate cost of punishment. Such consequences might be negative or positive. In the original corruption game, corruption stabilized cooperation by effectively offsetting the cost of punishment. There are, however, other ways in which this cost might be offset. A small benefit to punishers in interactions with cooperators, such as the status-payments described, could provide an alternative means to offset the cost of punishment. Here, we explore how such small benefits to punishers affect the maintenance of cooperation and the evolution of corruption and righteousness. Importantly, these payments avoid most components of a reputation system, where individuals decide whom to cooperate with based on information about past interactions received from others 47–49. Such reputation systems require cognitive and social resources that may be unavailable in some systems. Cooperators in our model automatically make small payments to punishers. For this to work, punishers need only be physically recognizable by cooperators or by a centralized authority. This is biologically plausible for social insects, where punishers are often larger and stronger [18], [19], [50]. In human societies, punishers can often be identified by cultural tags such as uniforms even in the absence of individual recognition, and payments can also be conferred via taxation systems without any need for individual observation.

## Methods

We consider an evolutionary game with four strategies, namely: cooperative non-punisher (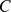), defecting non-punisher (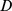), cooperative punisher (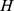) and defecting punisher (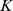). The game is defined by the payoff matrix
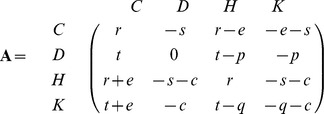
where each row corresponds to the four strategies in the above order. For conciseness, we will refer to the strategies as cooperator (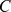), defector (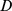), cooperative punisher (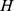), and defecting punisher (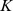). Throughout this article, we use bold letters to represent non-scalar variables with upper- and lower-case letters corresponding to matrices and vectors, respectively.

Parameters 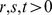 correspond to the payoffs of the Prisoner’s Dilemma where 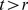. Traditionally, 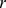 stands for the *reward* of cooperation, 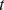 for the *temptation* of defection, and 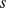 for the *sucker*’s payoff. For simplicity we assume that 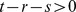. Parameters 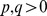 correspond to the cost experienced by a defecting non-punisher (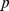) and a defecting punisher (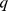) when punished. Parameter 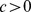 corresponds to the cost experienced by a punisher when punishing another individual. To account for payments from cooperators to punishers, we introduce the parameter 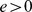. For simplicity, we will assume that 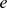 is very small, (in particular 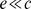). Although payment 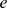 is made by non-punishers at an individual level during interactions, it is dynamically equivalent to a payment by all non-punishers.

Notice that in the absolute absence of defectors (or defecting punishers), cooperators have a smaller payoff than honest punishers which “solves” the problem of second-order free-riding. However, in the presence of even a small amount of defection (which is very biologically and socially realistic), punishers have a smaller payoff than pure cooperators. This is due to the fact that payments are very small (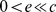) when compared to the costs of punishing.

There are two differences between our payoff matrix (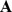) and the payoff matrix of the Corruption Game [20]: introduction of parameter 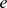, and collapsing the costs of inflicting a punishment on defectors (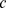) and on defecting punishers (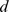) into one parameter (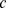). The choice to collapse 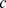 and 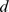 is to maintain tractability of the model by maintaining the same number of parameters. Moreover, the existence and stability of all equilibria in the Corruption Game was independent from 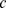 [20]. Although 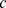 had a quantitative effect on the size of basins of attraction, parameter 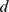 was also free in the sense that the relevant dynamics involved 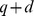, and never 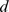 alone. Notice that in the special case when the cost of punishing is equal for both types of punishers (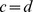) the Corruption Game corresponds to 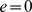.

The game reduced to only 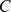 and 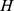 has a degenerate payoff matrix in the Corruption Game, that is, both strategies have exactly the same payoff 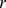. As a consequence, all equilibria consisting of cooperators and/or cooperative punishers are structurally unstable and were not analyzed by Úbeda and Duéñez-Guzmán [20]. In the current model, however, the game reduced to the strategies 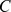 and 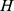 has a non-degenerate payoff matrix
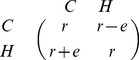
(1)thus avoiding the existence of structurally unstable equilibria.

We chose the perturbation in Equation (1) because of its simplicity and because it is zero-sum. Other perturbations can lead to qualitatively different dynamics, but require a significant surplus of payoffs (or costs), which is harder to justify biologically. For humans, 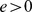 could result from increased social status of punishers; for other species, the value of 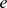 could be either positive or negative. When 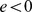, righteousness is globally unstable, and the dynamics lead to either defection or corruption, which is qualitatively equivalent to the Corruption Game. Thus, 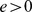 is the only situation which may lead to righteousness.

**Figure 1:**
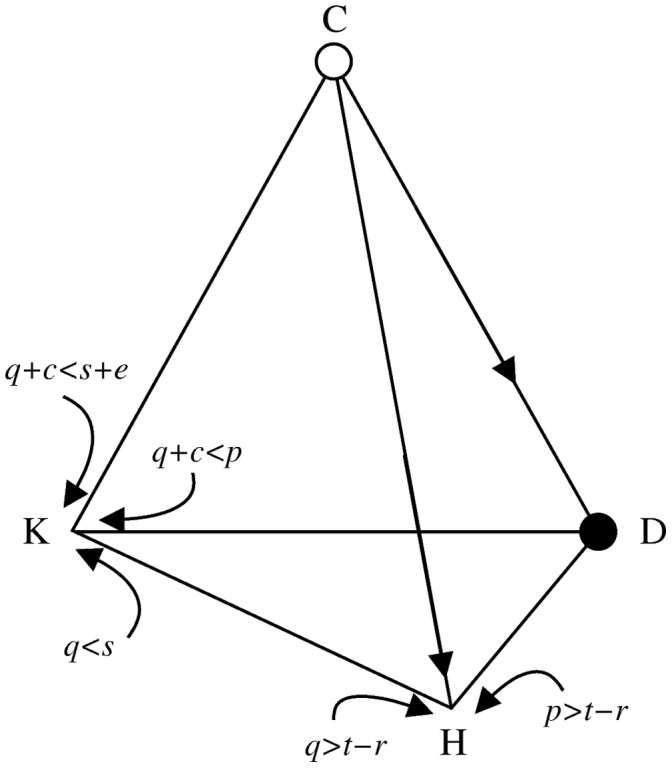
Conditions for stability of the four corners of the simplex. If the condition is satisfied, then the direction pointed by the arrow behaves as a local attractor. 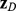 is always stable, denoted by the filled circle, while 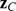 is always unstable, denoted by the open circle. While many equilibria at the edges of the simplex may be stable in the reduced games, we reserve filled circles to indicate globally stable equilibria (i.e. equilibria that are stable in the full game with the four strategies.).

Both cultural and genetic evolution are most commonly studied using *replicator dynamics* [51]–[53]. Like Úbeda and Duéñez-Guzmán [20], we analyze the model through the continuous time replicator dynamics equation:
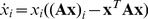
(2)where 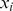 corresponds to the frequency of a strategy in the population, and subscript 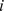 corresponds to each of the four strategies available 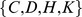. 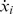 corresponds to the time derivative, and 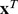 denotes the transpose of the column vector 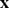. Note that we are representing the frequencies of strategies in the population by a vector 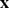 of dimension 4. Therefore, we can geometrically consider all possible population states as elements of the 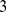-simplex. Populations consisting of only one strategy would lie at vertices of this tetrahedron (see Figure 1).

## Results

The equilibria of Equation (2) may rest at discrete points in the interior, corners, edges, or faces of the simplex formed by all possible population states. When an equilibrium is stable, it is locally asymptotically stable. We will refer to equilibria by 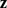 with subindices denoting where the equilibrium lies. For instance, 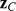 will correspond to the equilibrium at the vertex 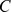, and 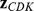 to an internal equilibrium in the face comprised by the strategies 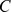, 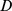 and 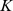. Note that this is a slight deviation from Úbeda and Duéñez-Guzmán [20] in which a different notation was used for equilibria at vertices, edges and faces of the simplex.

A monomorphic population of defectors (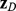) is always stable. As is customary, we will call this equilibrium *defection*. In addition to this equilibrium, two other stable equilibria can exist. One is either 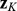 or 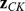 (i.e. a population comprised of defecting punishers or defecting punishers and cooperators), which we will refer to as *corruption*, and either 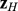 or 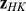 (i.e. a population comprised of cooperative punishers, perhaps with defecting punishers at low frequency) which we will refer to as *righteousness* (see Appendix).

**Figure 2:**
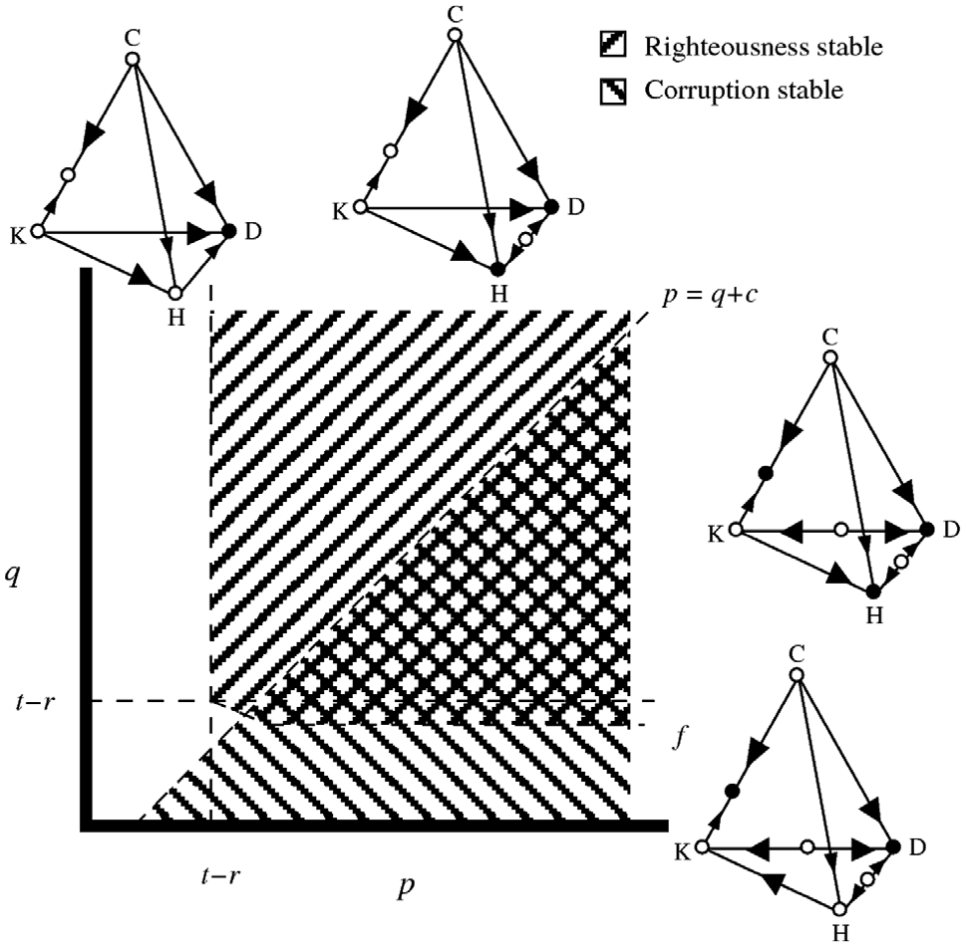
Stability of the three main equilibria on the system as a function of parameters 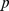 and 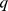. The white area corresponds to the cases in which defection is the only globally stable equilibrium. Notice that there is an area where righteousness and corruption intersect, in this region, all three main equilibria are stable. Depicted are representative cases for each of the four areas. While the position of the main equilibria might change and existence of other (unstable) internal equilibria in some edges might exist for specific parameter combinations, the qualitative dynamics are captured by these depicted cases. For simplicity, internal equilibria in the faces of the simplex are not drawn. All internal equilibria in the faces are unstable (see Appendix).



Figure 2 shows the conditions for stability of the three main equilibria (defection, corruption and righteousness) depending on the severity of the punishment towards defectors and corrupt punishers (parameters 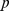 and 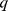, respectively). The total cost of punishing a corrupt punisher (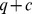) determines the stability of the corruption equilibrium. Corruption is stable whenever
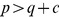
(3)either at 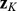 if 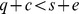 or at 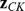 if 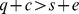, recalling that 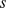 is the sucker’s payoff of cooperators against defectors, and 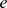 is the payment of cooperators to punishers. The overall temptation to defect (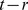, that is, the difference between the payoff of a defector and a cooperator against a cooperator) mediates the stability of the righteousness equilibrium. Righteousness is stable whenever

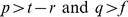
(4)that is, when the punishments for defection are severe enough to both defectors and corrupt punishers. Righteousness is stable either at 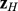 if 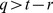 or at 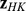 if 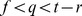; where 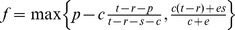 depends on 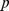 (see Text S1, equation S9), and is denoted by the two-segment line bounding the narrow region under 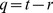 in Figure 2.

Note that the regions of stability for righteousness and corruption overlap all through the region delimited by 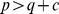 and 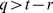, as well as through most of the narrow region where 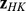 is stable (see Text S1). Intuitively, righteousness and corruption are both stable when punishment 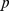 against non-punishers is larger than the total cost 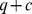 of punishing a punisher, and when the punishment 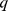 against corrupt punishers is severe (i.e. larger than the overall temptation to defect 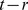). Recall that 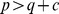 denotes a power inequality in favor of defecting punishers, and 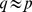 indicates a case of egalitarian punishments.

### Basins of Attraction: Simulations

To estimate the basin of attraction of each of the equilibria, we simulated the dynamics of the system numerically. All runs were performed with 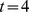, 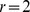, 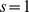, 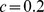 and 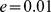.

We conduct the analysis for the punishment parameters 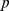 and 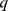 with values between 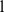 and 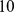 in increments of 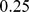. Given a value of the parameters 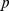 and 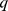, we analyze the dynamics starting close to the simplex corners 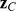, 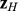 and 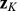. The corner 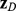 is not analyzed for it is always stable. For each of these three cases, we take a set of small perturbations (of order 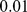) uniformly around the corresponding corner, and simulate the dynamical system using an Euler scheme until the population is close enough to one of the three main equilibria: defection, corruption or righteousness.

**Figure 3:**
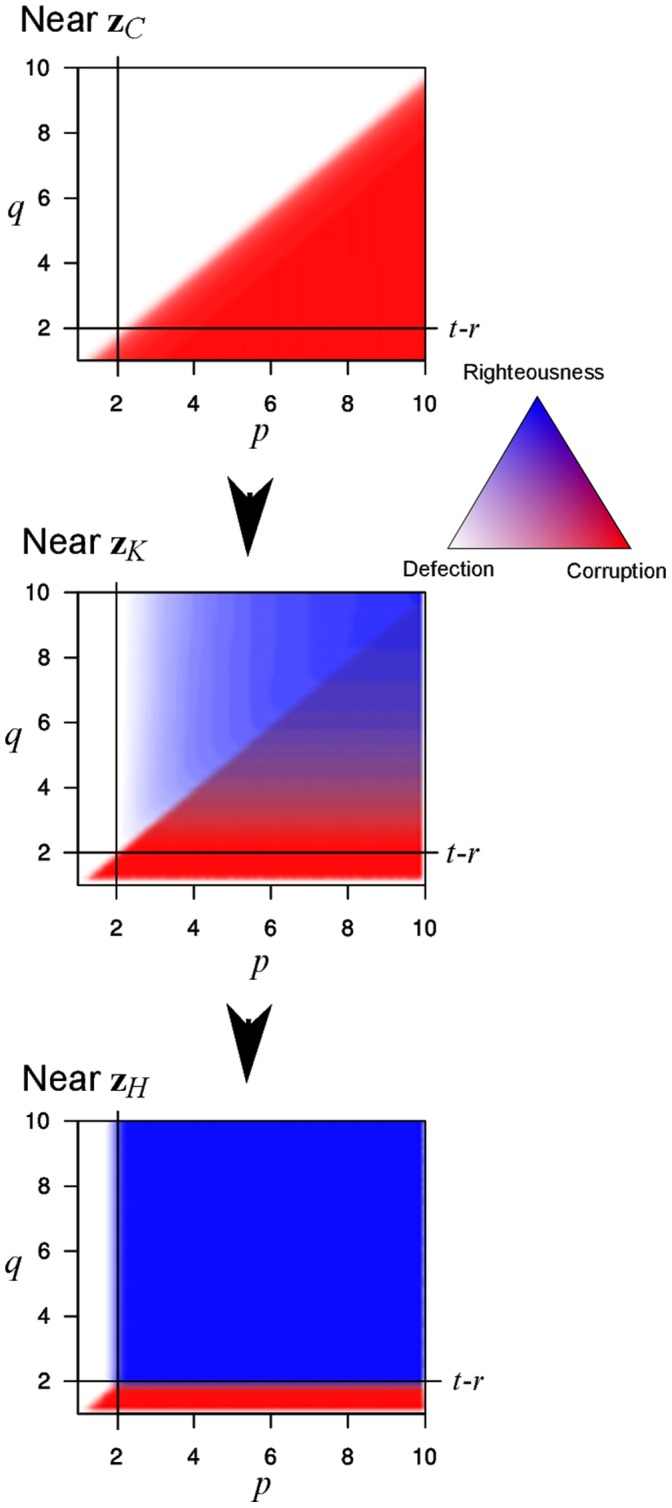
Dynamics of the system in the vicinity of 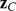 (top), 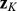 (middle) and 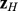 (bottom). The horizontal axis corresponds to the value of 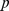. The vertical axis corresponds to the value of 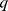. Isoclines represent the proportion of runs converging to corruption (red) and righteousness (blue). All runs that do not converge to either corruption or righteousness end up in defection (white).

We summarize the proportion of runs that end in each of the three possible equilibria (see Figure 3). These proportions are a numerical approximation of the equilibrium’s basin of attraction as a function of 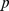 and 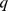.

As expected, whenever power inequalities favor non-punishers (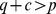, and thus corruption is unstable), the proportion of runs converging to corruption is zero (see Figure 2). In general, as long as 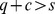, increasing power asymmetries (by increasing 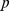 or decreasing 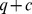) increases the basin of attraction of corruption. This is seen in Figure 3, where the basin of attraction of corruption increases from zero when 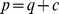 to close to one when 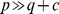.

It is worth noting that when the population starts at righteousness, and both 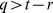 and 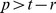, then the dynamics always remain at righteousness. Moreover, even when the population starts close to 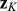, up to 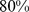 of the runs end up in righteousness (Figure 3). This proportion grows as both 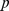 and 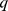 grow and is maximal whenever 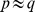, that is, when there are no power inequalities. This pattern is maintained even when the cost to punish 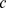 is much larger (data not shown).

## Discussion

We have explored the effect of a perturbation to the corruption game, namely, small payments (such as a slight increase in social status) to punishers. We find that the more egalitarian and harsher the punishments toward defectors and defecting punishers, the more likely the population will maintain cooperation through punishment and keep both corruption and defection at bay. In this scenario, the most likely outcome is a monomorphic population of cooperative punishers (righteousness). This shows not only that costly punishment can evolve (recall that 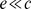), but that even when the social investment in punishment (a payment of 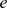 from non-punishing cooperators to punishers) diminishes as non-punishers shrink in frequency, punishing still pays off better than defecting. Moreover, the prevalence of cooperative punishers makes defection by punishers an inviable strategy, even when defecting punishers get more lenient punishments (power inequality). Thus, a righteous population can effectively resist the spread of corruption.

Righteousness, by stabilizing cooperation and providing a higher payoff to cooperative groups, constitutes a mechanism to shift the scale of selection from an individual to a group level. Unlike alternative mechanisms to maintain cooperation, such as reputation, righteousness requires no individual recognition or memory. Righteousness does require some ability to discriminate between punishers and non-punishers, but such discrimination can occur without complex cognition; for example, ant punishers are often larger and more aggressive than non-punishers [18], [19], [50].

Because the collective payoff of righteousness is higher than that of alternative outcomes, righteous groups are likely to outcompete those that have converged on defection or corruption. As a result, righteousness is expected to spread either culturally or genetically. This mechanism may explain the observation of righteous punishment in some ant species [21] and some human societies [54].

### The Path to Righteousness

Naïve cooperation is commonly taken as a starting point for studying the evolution of strategies in the Prisoner’s Dilemma and related games [2]. Our results show that a population that starts at or close to all cooperation will either go to defection or corruption, but not to righteousness (Figure 3, top). However, if it goes to corruption, it is possible to destabilize this equilibrium and have the population end up at righteousness in a reliable manner (Figure 3, middle and bottom). Much of the dynamics revolve around the costs imposed by corruption (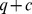) and the appeal of defection (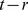).

An initially cooperating population that faces invasion by defectors might institute punishment in response. Even mild punishment (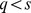, 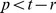) can suppress defection. However, the punishers in this population are susceptible to corruption. In fact, power inequalities that favor corruption (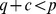) are required to keep defectors at bay, and if costs are small, corruption runs rampant (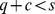).

Now that the population is corrupt, it is in a stable situation. Small changes to the costs and punishments will not change the dynamics qualitatively. While cooperation can be increased by increasing the cost of corruption, there will always be defecting punishers, and in fact they are needed to prevent defection spreading [20]. However, if cooperators invest even a tiny amount in punishment (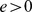), there is a possible route from corruption to righteousness. Conferring increased status on punishers, as occurs among humans, may be one form of such investment.

Corruption can be destabilized by making punishments both more egalitarian (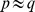) and harsher (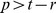 and 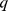 close to or above 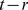, see inequality 4). Provided punishments are sufficiently harsh, completely removing power inequalities (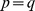) eliminates corruption, and cooperative punishment will likely spread. However, complete equality is likely to be unfeasible in human societies. Given that power inequalities cannot be removed completely, a sudden, large change in punishment can still destabilize corruption and stimulate a transition to righteousness.

The righteous population is resilient to invasion by both corruption and defection. In fact, righteousness is so stable that once there, a population needs to drop at least one of the punishments 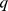 or 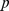 below 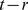 (making defection appealing once again) in order for a perturbation to destabilize it (see Figure 3, bottom). Power inequalities are largely irrelevant to the righteous population; reducing power inequalities is only required initially to destabilize corruption, and lead the dynamics toward righteousness instead of defection.

Mandatory payments from cooperators to punishers are justified empirically as discussed at the end of the introduction. However, we can conceive of a scenario in which there exist cooperators that do not make payments to punishers. In this case, we would have a line of neutral stability between the two types of cooperators as well between non-paying cooperators and honest punishers. Introducing this non-paying cooperator does not change the existence or stability of the righteousness equilibrium in the replicator equation. However, in the presence of noisy dynamics, righteousness could be lost due to drift (confirmed via numerical simulation; data not shown). This loss of righteousness is similar to the way tit-for-tat, which is an attractor and a promoter of cooperation in the presence of defection, is lost in noisy dynamics due to the neutral stability with pure cooperators [55]. Intuitively, righteousness is typically capable of eradicating defection from a population, which allows for non-paying cooperators to spread due to drift. Later, if no paying cooperators remain, the dynamics are governed by the original Corruption Game, and righteousness is lost.

Nonetheless, notice that the interpretation of the payment 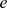 is flexible. For instance, assuming that pure cooperators have a small chance of giving a gift to a punisher instead of being it mandatory does not change the dynamics. In this way, 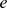 can be interpreted as the expected payment over many interactions.

### Consequences for Human Societies

Our results may help to explain the paradoxical data observed in human societies. Úbeda and Duéñez-Guzmán [20] suggested that if human cooperation is reliant on punishment, corruption should be universal among enforcers, negatively related to defection, and positively related to societal wellbeing. In reality, however, the extent of corruption varies markedly between societies and is negatively correlated with several aspects of economic development [27]–[29], [31] social wellbeing [23]–[26], [30] and cooperation [30]. Crime in general can be considered as defection, but corruption is positively related to other forms of crime [30].

In addition, whereas Úbeda and Duéñez-Guzmán [20] predict societies consisting of non-punishing cooperators governed by a corrupt minority, results from public goods games suggest that in some human societies, everybody punishes and most people cooperate [54]. Field studies of an egalitarian nomadic prestate society, the Turkana, also show that power inequalities are not required for the maintenance of large-scale cooperation via collective punishment of free-riders [56].

One possible explanation for the observed variance and negative impact of corruption is that some societies have transitioned, or are transitioning, from widespread corruption to righteousness (the reverse transition being much more difficult, as described above). Democratization and improved law enforcement may tend to reduce the power inequalities that favor corruption; such change may occur suddenly, facilitating the transition to righteousness, due to new policies or change of government. Because the total societal payoff of righteousness exceeds that of corruption, groups that have attained righteousness are likely to out-compete those that remain corrupt. For example, Mathew and Boyd [56] suggest that the cooperation generated by collective punishment may explain the dominance of the Turkana over competing groups.

For tractability, game theory models necessarily consider a restricted set of possible strategies. In contrast, humans may use an endless variety of strategies, including maladaptive ones such as antisocial punishment [14], [57]. More complex models, such as simulations incorporating more detailed social dynamics and complex strategies, can help to bridge the gap between analytical prediction and empirical reality. In this context, the current model suggests possible lines of research that could lead to significant policy reform.

Corruption is a major social problem, and its reduction is an active area of research. Our results suggest that social policy can stimulate such a transition by enforcing strong, egalitarian punishments. They also imply that without such policy change, corruption will remain ubiquitous. Intuitively, in trying to promote righteousness, it might seem appealing to punish corruption much more harshly than other forms of defection (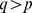). However, this does not maximize the chance of righteousness. Thus, in fighting corruption, a society should not yield to the temptation to overshoot power asymmetries from tolerating corruption (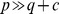) to severe reprisals against corruption (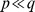). In other words, the path to righteousness starts with fairness, not with vengeance.

## Supporting Information

Text S1 **Analytical derivations of stability analysis in the righteousness game.**
(PDF)
