# Supplementary material for: Evolving Righteousness in a Corrupt World
Source: PLoS One. 2012 Sep 12;7(9):e44432. doi: 10.1371/journal.pone.0044432 (PMC3440392; doi:10.1371/journal.pone.0044432)
Supplement: Text S1 — Analytical derivations of stability analysis in the righteousness game. (PDF) [file pone.0044432.s001.pdf]

Supplementary Information: Evolving Righteousness in a Corrupt World

March 19, 2012

Analysis

We consider an evolutionary game with four strategies, namely: cooperative non-punisher (C), defecting non-punisher (D), cooperative punisher (H) and defecting punisher (K). The game is defined by the payoff matrix

$$\mathbf{A} \;=\; \begin{array}{c} \\ C \\ D \\ H \\ K \end{array} \begin{array}{c} \begin{array}{cccc} C & D & H & K \end{array} \\ \begin{pmatrix} r & -s & r-e & -e-s \\ t & 0 & t-p & -p \\ r+e & -s-c & r & -s-c \\ t+e & -c & t-q & -q-c \end{pmatrix} \end{array}$$

where each row corresponds to the four strategies in the above order. For conciseness, we will refer to the strategies as cooperator (C), defector (D), cooperative punisher (H), and defecting punisher (K). Throughout this article, we use bold letters to represent non-scalar variables with upper- and lower-case letters corresponding to matrices and vectors, respectively.

Like Úbeda & Duéñez Guzmán (2011), we analyze the model through the continuous time *replicator dynamics* (Hofbauer & Sigmund, 1998):

$$\dot{x_i} = x_i((\mathbf{A}\mathbf{x})_i - \mathbf{x}^T\mathbf{A}\mathbf{x}) \tag{S1}$$

Equilibria and Stability

Interior

An equilibrium population $\mathbf{x}$ in which no strategy has gone to exticntion satisfies

$$(\mathbf{Ax})_C = (\mathbf{Ax})_D = (\mathbf{Ax})_H = (\mathbf{Ax})_K. \tag{S2}$$

Therefore, internal equilibria are normalized solutions to the system $\mathbf{Ax} = \mathbf{1}_4$, where $\mathbf{1}_n$ is the vector of all ones of size n.

Solving the linear system and assuming $e^2 = 0$, we find that the equilibrium exists if an only if

$$\mathbf{y}_{CDHK} = \frac{1}{est}\begin{pmatrix} -c(t-r) \\ (c-e)(t-r)+es \\ c(t-r)+es \\ -c(t-r)-es \end{pmatrix} \tag{S3}$$

can lie inside the simplex. However, the components have opposite signs and therefore $\mathbf{y}_{CDHK}$ cannot lie in the simplex. Therefore, all equilibria must lie in the corners, edges, or faces of the simplex, and, by the exclusion principle (Hardin, 1960), all interior trajectories converge to the simplex boundary.

The normalization factor $1/est$ is so that the sum of the coordinates of $\mathbf{y}_{CDHK}$ adds up to one (i.e. so it lies on the simplex). To streamline notation we will ommit this normalization factor, and instead give the values of $\mathbf{y}$ as proportional to a vector, using the notation

$$\mathbf{y}_{CDHK} \propto \begin{pmatrix} -c(t-r) \\ (c-e)(t-r)+es \\ c(t-r)+es \\ -c(t-r)-es \end{pmatrix}$$

Corners

The corners of the simplex are always equilibria (see Equation S1): $\mathbf{z}_C = (1,0,0,0)^T$, $\mathbf{z}_D = (0,1,0,0)^T$, $\mathbf{z}_H = (0,0,1,0)^T$, $\mathbf{z}_K = (0,0,0,1)^T$.

We analyze the local stability of each strategy using the eigenvalues of the Jacobian matrix of the system (Equation S1) evaluated at each corner.

It is worth noting that for any frequency based analysis of n strategies, we only need to consider $n-1$ eigenvalues of the Jacobian, since there will always be an eigenvector that lies outside of the simplex (i.e. the eigenvector $\mathbf{v}$ such that $\mathbf{v}^T.\mathbf{1} \neq 0$). For this reason, whenever we discuss eigenvalues of Jacobians, we will only give those that are associated with eigenvectors laying in the simplex.

The eigenvalues for the corner equilibria $\mathbf{z}_C$, $\mathbf{z}_D$, $\mathbf{z}_H$ and $\mathbf{z}_K$ are

$$\{t-r, e, t-r+e\}, \qquad \{-s, -s-c, -c\},$$

$$\{-e, t-r-p, t-r-q\}, \quad \{q+c-s-e, q+c-p, q-s\}.$$

respectively.

It is now possible to analyze local stability of these equilibria. Only $\mathbf{z}_D$ is always stable, and $\mathbf{z}_C$ is always unstable (recall that $t > r$). $\mathbf{z}_H$ can be stable in the directions pointing towards $\mathbf{z}_D$ and $\mathbf{z}_K$ if $p > t-r$ and $q > t-r$ respectively; as discussed above, $\mathbf{z}_H$ is stable in the direction pointing towards $\mathbf{z}_C$ if and only if $e > 0$. Finally, if $q+c < s+e$, $q+c < p$ and $q < s$, then $\mathbf{z}_K$ is stable in the directions of $\mathbf{z}_C$, $\mathbf{z}_D$ and $\mathbf{z}_H$ respectively.

Edges

For convenience we will use $\mathbf{y}$ to represent an equilibrium in a reduced game, much in the same way as we use $\mathbf{z}$. The difference here being that $\mathbf{y}$ will have as many components as strategies are in the reduced games, while $\mathbf{z}$ is always a vector with 4 components. There is a natural mapping from a $\mathbf{y}$ vector to a $\mathbf{z}$ vector, simply by inserting zeroes in the components of $\mathbf{z}$ that are not in its subindex (i.e. not present in $\mathbf{y}$). For example $\mathbf{y}_{DH} = (y_1, y_2)$ corresponds to an internal equilibrium in the reduced game between D and H, and $\mathbf{z}_{DH} = (0, y_1, y_2, 0)$ corresponds to that same equilibrium, but considered in the whole game.

Existence of internal equilibria in reduced games with two strategies are as in (Úbeda & Duéñez Guzmán, 2011), except for the game between C and H. Conditions for stability are slightly different, and the derivations are explicitly given whenever they differ from (Úbeda & Duéñez Guzmán, 2011). For completeness, we summarize the conditions for existence and stability of all two-strategy games below.

Game Between C and D This case corresponds to the well-known Prisoner's Dilemma.

Game Between C and H This game is characterized by the payoff matrix

$$\mathbf{A}_{CH} = \begin{pmatrix} r & r-e \\ r+e & r \end{pmatrix} \tag{S4}$$

which corresponds to a perturbation of the neutral line of stability in the original Corruption Game.

An internal equilibrium exists if and only if the solution $\mathbf{y}_{CH}$ of the system $\mathbf{A}_{CH}\mathbf{y}_{CH} = \mathbf{1}_2$ can lie on the interior of the simplex. However,

$$\mathbf{y}_{CH} \propto \begin{pmatrix} \frac{1}{e} \\ -\frac{1}{e} \end{pmatrix}$$

is the solution to that system, and therefore it cannot lie in the simplex. Consequently, there is no internal equilibrium.

Game Between C **and** K This game has an interior equilibrium of the form

$$\mathbf{y}_{CK} \propto \begin{pmatrix} q+c-s-e \\ t-r+e \end{pmatrix} \tag{S5}$$

if and only if $q + c > s + e$.

Equilibrium $\mathbf{z}_{CK}$ is stable in the whole game if and only if all these are satisfied

$$\begin{aligned} -(t-r+e)(q+c-s-e) &< 0 \\ (q+c-p)(t-r)+e(e+s-p) &< 0 \\ e(t-r-s+q)-c(t-r) &< 0 \end{aligned}$$

That is, if $\mathbf{z}_K$ is stable from the direction of $\mathbf{z}_D$ $(p > q + c)$, K is unstable toward $\mathbf{z}_C$ $(q + c > s + e)$, and e is small.

Game Between D **and** H This game has an interior equilibrium of the form

$$\mathbf{y}_{DH} \propto \begin{pmatrix} p+r-t \\ s+c \end{pmatrix} \tag{S6}$$

if and only if $\mathbf{z}_H$ is stable from the direction of $D(p > t - r)$.

The equilibrium $\mathbf{z}_{DH}$ is always unstable in the full game.

Game Between D **and** K This game has an interior equilibrium of the form

$$\mathbf{y}_{DK} \propto \begin{pmatrix} p-q-c \\ c \end{pmatrix} \tag{S7}$$

if and only if $\mathbf{z}_K$ is stable from the direction of $\mathbf{z}_D$ $(p > q + c)$.

The equilibrium $\mathbf{z}_{DK}$ is always unstable in the full game.

Game Between H and K There is an interior equilibrium

$$\mathbf{y}_{HK} \propto \begin{pmatrix} q-s \\ t-r-q \end{pmatrix} \tag{S8}$$

if and only if $t-r > q > s$.

The equilibrium $\mathbf{z}_{HK}$ is stable if and only if all of the following are satisfied

$$\begin{aligned} -(q-s)(t-r-q) &< 0 \\ c(t-r-q) - e(q-s) &< 0 \\ c(t-r-q) - (t-r-s)(p-q) &< 0 \end{aligned} \tag{S9}$$

In particular, this implies that q is very close to $t-r$, we also require $e > 0$ and $p > q$. Therefore, we can parametrize the conditions in the above inequalities as

$$\begin{aligned} t-r &> q > s \\ p > q &> \max\left\{\rho\left(1+\frac{c}{t-r-s-c}\right), e\frac{(t-r-s)}{c+e}\right\} \end{aligned}$$

where $\rho = t-r-p$.

Moreover, if $\mathbf{z}_{CK}$ is stable, then $p > q + c$ which implies that $p - q > c$, and thus

$$-(t-r-s)(p-q) < -c(t-r-s) < 0$$

so this inequality is automatically satisfied.

In other words, both $\mathbf{z}_{HK}$ and $\mathbf{z}_{CK}$ can be globally stable.

Faces

Here we focus on the three-strategy games, and analyze existence of internal equilibria as well as their stability.

Game without C This game has an internal equilibrium of the form

$$\mathbf{y}_{DHK} \propto \begin{pmatrix} (t-r)(q+c-p) - (s(p-q)-cq) \\ s(p-q)-cq \\ c(t-r) - (s(p-q)-cq) \end{pmatrix} \tag{S10}$$

which exists if and only if $s(p-q) > cq$, $c(t-r) > s(p-q) - cq$, and $(t-r)(q+c-p) > s(p-q) - cq$. In particular, $q + c > p > q$ is required.

The equilibrium $\mathbf{z}_{DHK}$ is stable if and only if the following conditions are satisfied

$$\begin{array}{rcl} c(p-q)(t-r)+e\left(s(p-q)-cq\right) & < & 0 \\ \chi\left(s(p-q)-c(q+c)\right)+c(p-q)\sigma \pm \sqrt{\Delta} & < & 0 \end{array}$$

where $\chi = t-r-q$, $\sigma = t-r-s$ and Δ is an expression that depends on the parameters of the game.

Observe that since $e \approx 0$, then $c(p-q)(t-r)+e\left(s(p-q)-cq\right) > 0$ and therefore the equilibrium is always unstable.

Game without D If an internal equilibrium existed, it would be of the form

$$\mathbf{y}_{CHK} \propto \left(\begin{array}{c} cq-(c-e)(t-r)-es \\ -eq+(c-e)(t-r)+es \\ eq \end{array}\right) \tag{S11}$$

Which is an interior point when H is stable from K ($q > t-r$) and e is small enough.

Let $\alpha = (c-e)(t-r)+es$, and note that $cq > \alpha > eq$ for the equilibrium to exist. The equilibrium $\mathbf{z}_{CHK}$ is stable if and only if

$$\begin{array}{rcl} (q-p+e)\alpha & < & 0 \\ \beta+\sqrt{\beta^2-4\gamma} & < & 0 \\ \beta-\sqrt{\beta^2-4\gamma} & < & 0 \end{array}$$

where $\beta = \alpha - q(q+c-s)$ and $\gamma = eq(eq-\alpha)(cq-\alpha)$. Notice that if $e > 0$, then $-4\gamma > 0$ and thus, the discriminant $\sqrt{\beta^2-4\gamma}$ is larger than $|\beta|$. Therefore, it is impossible for all quantities to be negative at the same time, and then equilibrium $\mathbf{z}_{CHK}$ is always unstable.

Game without H This game has an internal equilibrium of the form

$$\mathbf{y}_{CDK} \propto \left(\begin{array}{c} c(p-e)-(p-q)s \\ e(p-e-s)-(q+c-p)(t-r) \\ c(t-r)+es \end{array}\right) \tag{S12}$$

which, given that $e \approx 0$, exists if and only if $p > q+c$ and $(p-q)s < c(p-e)$.

As before, let $\alpha = (c-e)(t-r)+es$. Equilibrium $\mathbf{z}_{CDK}$ is stable if and only if the following inequalities hold

$$\begin{aligned} \alpha(q+e-p) &< 0 \\ \tilde{\beta} \pm \sqrt{\tilde{\beta}^2 + 4\tilde{\gamma}} &< 0 \end{aligned}$$

where $\tilde{\beta} = c(t-r)(q+c-e) - ((t-r+e)(p-q)-ce)s$ and

$$\tilde{\gamma} = (c(t-r)+es)(c(p-e)-s(p-q))(e(p-e-s)+(p-q-c)(t-r))$$

Nota that each factor of $\tilde{\gamma}$ is positive, thus, the discriminant is larger than $\tilde{\beta}$, which implies that not all eivengalues can be negative at the same time. Therefore, equilibrium $\mathbf{z}_{CDK}$ is always unstable.

Game without K This game corresponds to the Punishment Game and has an internal equilibrium of the form

$$\mathbf{y}_{CDH} \propto \begin{pmatrix} -c(t-r-p)-e(c+s) \\ e(p-e) \\ c(t-r)+es \end{pmatrix} \tag{S13}$$

which, given that $e \approx 0$, we can assume $p > |e|$ and $c > |e|$. In this case, $(c+e)(p-e) > 0$, and the equilibrium exists if and only if $-e(c+s) > c(t-r-p)$. In particular, in order for the equilibrium to exist, if $e > 0$, then $p > t-r$, and if $e < 0$, it requires p to be not too much smaller than $t-r$.

Equilibrium $\mathbf{z}_{CDH}$ is stable if and only if the following condition are satisfied

$$\begin{aligned} (p-q-e)(c(t-r)+es) &< 0 \\ e\,(c(t-r)+ps) \pm \sqrt{\Delta} &< 0 \end{aligned}$$

where Δ is an expression that depends on the parameters of the game.

If $e > 0$, then $e\,(c(t-r)+ps) > 0$ which makes equilibrium $\mathbf{z}_{CDH}$ always unstable.

References

Hardin, G. 1960. The Competitive Exclusion Principle. *Science*, **131**, 1292–1297.

Hofbauer, Josef, & Sigmund, Karl. 1998. *Evolutionary Games and Population Dynamics*. Cambridge University Press.

Úbeda, Francisco, & Duéñez Guzmán, Edgar A. 2011. Power and Corruption. *Evolution*, **65**(4), 1127–1139.
